# The potential role of mother-in-law in prevention of mother-to-child transmission of HIV: a mixed methods study from the Kilimanjaro region, northern Tanzania

**DOI:** 10.1186/1471-2458-11-551

**Published:** 2011-07-12

**Authors:** Eli Fjeld Falnes, Karen Marie Moland, Thorkild Tylleskär, Marina Manuela de Paoli, Sebalda Charles Leshabari, Ingunn MS Engebretsen

**Affiliations:** 1 Centre for International Health, University of Bergen, Postboks 7804, N-5020 Bergen, Norway; 2 Faculty of Health and Social Sciences, Bergen University College, Postboks 7030, N-5020 Bergen, Norway; 3 Fafo Institute for Applied International Studies, Postboks 2947, Tøyen, N-0608 Oslo, Norway; 4 School of Nursing, Muhimbili University College of Health Sciences, PO Box 65001, Dar es Salaam, Tanzania

## Abstract

**Background:**

In the Kilimanjaro region the mother-in-law has traditionally had an important role in matters related to reproduction and childcare. The aim of this study was to explore the role of the mothers-in-law in prevention of mother-to-child transmission (PMTCT) service utilization and adherence to infant feeding guidelines.

**Methods:**

The study was conducted during 2007-2008 in rural and urban areas of Moshi district in the Kilimanjaro region of Tanzania. Mixed methods were used and included focus group discussions with mothers-in-law, mothers and fathers; in-depth interviews with mothers-in-law, mothers, fathers and HIV-infected mothers, and a survey of 446 mothers bringing their four-week-old infants for immunisation at five reproductive and child health clinics.

**Results:**

The study demonstrated that the mother-in-law saw herself as responsible for family health issues in general and child care in particular. However she received limited trust, and couples, in particular couples living in urban areas, tended to exclude her from decisions related to childbearing and infant feeding. Mothers-in-law expected their daughters-in-law to breastfeed in a customary manner and were generally negative towards the infant feeding methods recommended for HIV-infected mothers; exclusive replacement feeding and exclusive breastfeeding.

**Conclusions:**

Decreasing influence of the mother-in-law and increasing prominence of the conjugal couples in issues related to reproduction and child care, reinforce the importance of continued efforts to include male partners in the PMTCT programme. The potential for involving mothers-in-law in the infant feeding component, where she still has influence in some areas, should be further explored.

## Background

There is increasing awareness of the importance of male partner involvement in prevention of mother-to-child transmission of HIV (PMTCT) programmes [1,2], but little attention has been paid to the role of the mother-in-law [3]. Key interventions in the PMTCT programmes include counselling and testing for HIV at the antenatal clinic, drug treatment and counselling on safer infant feeding practises. The HIV-infected mother is expected to make decisions on these complex and sensitive issues in an autonomous manner. However, it has been shown that these decisions are not taken in a social vacuum [4]. The male partner is influential, but also other people surrounding the mother may affect her decisions [4-6]. Several studies carried out in sub-Saharan Africa, and from patrilinear societies in particular, have demonstrated the important role of the mother-in-law, i.e. the paternal grandmother, in infant feeding and infant care after delivery [3,5,7-11]. Women who are confronted with conflicting advice from health workers and their mother-in-law often find it difficult to ignore the authority of the mother-in-law [8]. Studies carried out in Tanzania and the Ivory Coast reported that the mother-in-law was an obstacle to safe infant feeding practises through exclusive breastfeeding or exclusive replacement feeding for HIV-infected mothers [7,9].

In Tanzania, the estimated prevalence of HIV in pregnant women attending antenatal care during 2007 was 8.2% [12]. The PMTCT programme was piloted from 2000 [13], and was thereafter expanded country-wide [14]. The first national PMTCT guidelines, issued in 2004, were adhered to during this study period [15]. The infant feeding guidelines included were in accordance with the 2001 WHO guidelines [16]. The mothers were advised on three infant feeding options: (a) exclusive breastfeeding for six months or early cessation any time convenient to the individual woman's situation, (b) replacement feeding with commercial infant formula, and (c) replacement feeding with home modified cow's milk.

Few studies have focused on the role of the mother-in-law for service utilization and adherence among women enrolled in the PMTCT programmes. Hence, the potential for involvement of mother-in-law as a significant other in PMTCT is largely unexplored. This study focused on the relationship between mothers-and daughters-in-law in the Kilimanjaro region and aimed to explore the influence of mothers-in-law on PMTCT service utilization. More specifically the study sought to explore: 1) and 2) the potential for the participation of mothers-in-law in the promotion and support of safe infant feeding practices.

## Methods

### Study setting

The study was conducted from October 2007 to February 2008 at five reproductive and child health clinics in urban and rural areas of Moshi district in the Kilimanjaro region of north-eastern Tanzania. HIV counselling and testing was offered routinely as part of the antenatal care in each of the participating clinics. More details of the PMTCT programme in the region has been described elsewhere [17].

The region has high antenatal service participation (99%), relatively high rates of women giving birth in a health facility (70%) and a population with relatively high education (64.9% of women completed primary school) [18]. Breastfeeding is universal and 98.4% of children in this region have been breastfed [18]. A mixed feeding pattern, however, with early introduction of water, other fluids and porridge in addition to breast milk, is commonly practised [19].

The Kilimanjaro region is multi-ethnic, but the Chagga people constitute the dominant ethnic group. They are known to be well educated and to utilize public health services more extensively than other ethnic groups in the region [10,20]. Among the Chagga and in the Kilimanjaro region as a whole, patrilinear kinship system still prevails and patrilocal residence is to some extent still practised, particularly in rural areas. After marriage, the woman moves to the homestead of the husband and his parents. With marriage traditionally being viewed as a contract between the two lineages, the conjugal union has been relatively weak [10,21]. The husbands' parents play an important role in decisions related to reproduction and family health [21]. This places a married woman in a position of dependence on her affinal kin, particularly her mother-in-law. In this region, and in particular among the Chagga people, the confinement period after birth has been a highly appreciated period of rest. During this period the daughter-in-law customarily lives together with her mother-in-law, who has a responsibility to care for her, ensuring that she consumes enough nutritious food to produce sufficient milk [10]. The privileges enjoyed by the postnatal mother are closely related to her breastfeeding the child [10].

### Mixed methods

**Figure 1:**
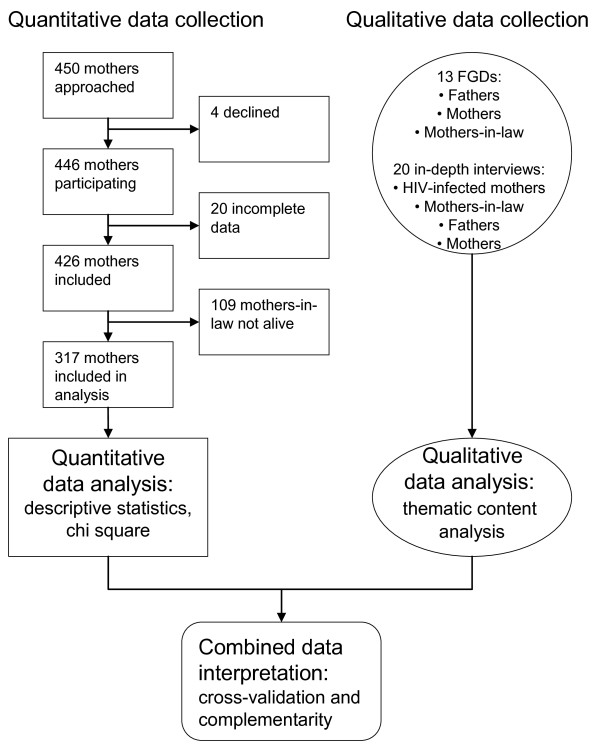
**Mixed methods: concurrent triangulation**.

This study forms part of a study on the mother's utilization of the PMTCT services and the methods employed has been described in detail elsewhere [22]. Briefly, mixed methods with a concurrent triangulation design was utilized [23] (Figure 1). A cross sectional survey was conducted concurrently with qualitative in-depth interviews and focus group discussions (FGDs). In the study described in this paper, the quantitative data served to complement the qualitative data obtained. The quantitative and qualitative data were analysed separately and were first integrated during interpretation of the results.

### Quantitative data

The five clinics were purposely selected to represent urban and rural areas of Moshi and to enable follow-up of previous research carried out in the same sites [6,9,11]. This provided the opportunity to compare the results with previous findings in the same area about the role of the mother-in-law. One of the urban clinics was part of a pilot of the PMTCT programme from 2000; the other two urban clinics implemented PMTCT in 2004, and the two rural clinics implemented the programme in 2006. In our previous study on the mother's utilization of the PMTCT services, including the same five clinics, there was no significant (p < 0.05) difference between the urban and rural antenatal attendees with regards to PMTCT practices, i.e. receiving counselling and testing [22]. During the data collection period every mother who attended one of the five participating clinics with their infant for the first doses of the diphtheria, pertussis, tetanus, hepatitis B (DPT-HB) and polio immunisation, was invited to take part in the study. This first time immunization has a coverage of 100% in the region [18]. Nursing staff at the clinics was informed about the purpose of the study, and explained it to each mother before asking for their participation. In total, 450 mothers were approached; 446 (99%) agreed to participate. Of these, 20 were excluded from the data analysis owing to incomplete data; of the 426 remaining mothers, 317 reported having a live mother-in-law. These were included in the final data analysis (Figure 1). More details on the survey has been described elsewhere [22].

Four female research assistants conducted the interviews. The most senior of them, a retired nurse with extensive experience in mother- and child health, served as coordinator. She had previously served as the main research assistant in one of the former studies on PMTCT and infant feeding in the area [6,19] and had experience in conducting quantitative and qualitative interviews. The questionnaire was designed to collate information related to socio-demographic characteristics, clinic attendance, birth, infant feeding, counselling and testing for HIV, knowledge about PMTCT, relationships with the male partner and the mother-in-law. Information about HIV status was not collected.

The questionnaire included six questions concerning the mother's relationship with her partner and six similarly phrased questions concerning her relationship with her mother-in-law. The Visual Analogue Scale (VAS) was used to collect these data [24]. VAS is an instrument intended to measure a characteristic or attitude that is believed to range across a continuum of values that cannot easily be directly measured [25]. The ends of the scale are defined as the extreme limits (worst and best) of the parameter to be measured orientated from the left (value zero) to the right (value ten). In this study, zero represented "I do not agree at all" and ten represented "I totally agree". The mothers moved the marker to the position accordingly. They were given a thorough explanation of the scale beforehand and two test questions were used to illustrate the scale and assess their understanding of it.

Data were double entered into Epidata 3.1 software (http://www.epidata.dk) and analysed using SPSS PASW. We used descriptive statistics to assess categorical baseline characteristics. For each of the questions concerning the mother's relationship to her mother-in-law and partner the mean scores were calculated with 95% confidence intervals (CI). The mean scores of the questions concerning her mother-in-law were compared with the mean scores of the questions concerning her partner using a paired t-test.

### Qualitative data

The mothers-in-law, mothers and fathers in the FGDs and in-depth interviews were recruited from various villages in urban and rural Moshi. The villages were within the catchment areas of the clinics included in the quantitative study. Participants with children/grandchildren less than one year were purposively selected assuming that they were enrolled in or had been exposed to the PMTCT programme in the last pregnancy of the mothers. The participants were recruited by the main research assistant, her acquaintances in the respective villages and village leaders. Except from the five purposely selected HIV-infected mothers, the researchers did not know the HIV status of any of the participants.

A semi-structured interview guide was prepared for each group of participants. Themes included in the FGD and in-depth interview guides for the mothers-in-law were their knowledge of MTCT and its prevention, their attitude towards the PMTCT programme, their role in child health care and infant feeding, perceived reactions to non-breastfeeding and exclusive breastfeeding, their relationship to their daughter-in-law and their reactions to a hypothetically HIV-infected daughter-in-law. Individual informed consent was obtained after the participants had been informed of the study objectives.

Thirteen FGDs were conducted. Four were conducted with mothers-in-law, four with mothers and five with fathers. Employing FGDs allowed group interaction, which could help people to explore and clarify their views in a different manner than in one-to-one interviews [26]. The FGDs had between five and twelve participants in each group. The venues for the FGDs were chosen according to the convenience of the study participants. All were conducted outdoors, in a private compound, in a church or in a school building. The different venues did not seem to influence the flow of the discussions and degree of disclosure. The FGDs were moderated by a nurse working at a local HIV organization. She had training and experience in conducting FGDs and experience of talking with people about HIV. The discussions were conducted in Swahili.

Twenty in-depth interviews were conducted. Five of these took place with mothers-in-law, five with mothers of unknown HIV status, five with HIV-infected mothers and five with fathers. The aims of the in-depth interviews were to gain a deeper insight into expectations and experiences to the role of the mother-in-law with regards to PMTCT. Two of the HIV-infected mothers were recruited through a local HIV organization and three with the help of the main research assistant. The HIV-infected mothers had all participated in a PMTCT programme during their last pregnancy. The mothers, fathers and mothers-in-law were interviewed in their homes. The HIV-infected mothers were interviewed in settings chosen by themselves, to reduce potential discomfort and risk of disclosure. In order to be able to ask follow-up questions and to probe whenever necessary, all in-depth interviews were carried out by the principal investigator using the main research assistant as an interpreter. She was fluent in English and Swahili and the main local languages.

The FGDs and the in-depth interviews took between 45-90 minutes to complete. They were recorded with the consent of the participants and subsequently transcribed verbatim. Interviews conducted in Swahili were translated into English.

#### Qualitative data analysis

Qualitative data were analysed by the principal investigator in collaboration with the co-authors. A thematic content approach, which was guided by the Graneheim and Lundman framework, was utilized [27]. The material was systematically read through, line by line, in order to identify the meaning units. Meaning units were defined as string of the text that expressed a single coherent thought, up to the point at which the coherent thought changed. The meaning units were coded using a describing cue related to what the text bit concerned, e.g. 'role of the mother-in-law'. Each of the codes were organised so that codes concerning the same subject were grouped together into categories. The interview guide was used as a point of departure for grouping information, but during the analysis new categories were developed, e.g. from the category 'relationship to mother-in-law' to the category 'lack of trust'. The underlying meaning of the categories was formulated into a theme, e.g. the theme 'increasing tensions'. Further examples of the analysis include: the category 'attitudes to non-breastfeeding' was formulated into the theme 'expectations to breastfeed' and the category 'disclosure of HIV status' were formulated into the theme 'disclosure and support'. The information obtained in the in-depth interviews and FGDs was analysed and merged according to the codes and themes. Illustrative quotations were selected. Original data were re-assessed by the principal investigator and one of the co-authors after analysis in order to detect any concepts or information that had been missed and to meet consensus of opinion between the analysts.

### Ethics

The study was approved by the National Institute for Medical Research Tanzania, the Tanzanian Commission for Science and Technology, the Kilimanjaro Christian Medical Centre Ethical Research Committee and the Regional Committees for Medical and Health Research Ethics for Region West, Norway. All participants provided individual informed consent.

## Results

The results are presented in two parts. The first part concerns the role of the mother-in-law in the family, particularly after childbirth. The second part concerns her role in PMTCT, particularly with regard to infant feeding.

### Quantitative sample characteristic

Almost half the mothers lived in a rural area, and the majority (90.9%) were married or cohabiting (Table 1). The most common ethnic group was Chagga (60.6%). The father of the child was usually reported to be the head of the household (86.4%).

**Table 1:** Socio-demographic characteristics of women attending reproductive and child health clinics for childhood immunizations

Background factor	N = 317	Total (%)
Residence		
Rural	137	(43.2)
Urban	180	(56.8)
Mothers age, y		
< = 25	163	(51.4)
> 25	154	(48.6)
Marital status		
Married/cohabiting	288	(90.9)
Single/divorced/widow	29	(9.1)
Religion		
Catholic	140	(44.2)
Protestant	120	(37.9)
Muslim/other	57	(18.0)
Ethnicity		
Chagga	192	(60.6)
Pare/other	125	(39.4)
Education, mother		
0-7	165	(52.1)
8 +	152	(47.9)
Head of household		
Father of the child	274	(86.4)
Other	43	(13.6)

### The customary role of the mother-in-law

#### Responsibility after birth

Mothers living in a rural area were more likely to live with their mother-in-law than mothers living in an urban area (25.5% vs. 6.1%; p < 0.001). They were also more likely to report to meet their mother-in-law at least once a week (59.9% vs. 28.3%; p < 0.001) (Table 2). Approximately one third of mothers reported that they had moved in with their mother-in-law after giving birth to their last born child.

**Table 2:** The mother-in-law: living proximity and decision making in rural and urban areas

	All N = 317 n (%)	Rural N = 137 n (%)	Urban N = 180 n (%)	
The mother-in-law lives				
Together with the couple	46 (14.5)	35 (25.5)	11 (6.1)	***
Same/nearby village	142 (44.8)	54 (39.4)	88 (48.9)	
Far away	129 (40.7)	48 (35.0)	81 (45.0)	
Meet the mother-in-law				
> Once a week	133 (42.0)	82 (59.9)	51 (28.3)	***
< Once a week	184 (58.0)	55 (40.1)	129 (71.7)	
Moved to the mother-in-law after birth				
Yes	115 (36.3)	49 (35.8)	66 (36.7)	
No	202 (63.7)	88 (64.2)	114 (63.3)	
The mother-in-law makes the decision on				
Clinical attendance	1 (0.3)	1 (0.7)	0 (0.0)	
Family planning	2 (0.6)	1 (0.7)	1 (0.6)	
HIV testing	1 (0.3)	1 (0.7)	0 (0.0)	
Infant feeding	6 (1.9)	6 (4.4)	0 (0.0)	
Primary confidant				
Male partner	191 (60.3)	81 (59.1)	110 (61.1)	
Mother	59 (18.6)	21 (15.3)	38 (21.1)	
Mother-in-law	4 (1.3)	4 (2.9)	0 (0.0)	
Sister/other	63 (19.8)	31 (22.6)	32 (17.7)	

*** p < 0.001.

Qualitative interviews confirmed that the custom of a daughter-in-law staying in the house of her mother-in-law after giving birth was still practised, particularly in rural areas. A grandchild was seen to belong to the lineage of the father. This gave the mother-in-law rights and obligations concerning her paternal grandchild. Her main responsibility was to make sure that the daughter-in-law was capable of caring for her grandchild, the new member of the lineage. Among the mothers and fathers, there were diverging opinions about the customary role of the mother-in-law. Some rural mothers and fathers expressed that they saw the role of the mother-in-law after childbirth as important.

We send our wives to the village or have our mothers come to our home because there are things that are required that she must do for her daughter-in-law, like massaging and cooking. These are things that I cannot do. (Rural father, FGD)

However, there was a tendency among urban couples to express a more independent position, evading this practice. Some fathers stated that they as fathers should take the responsibility customarily assigned to the mother-in-law.

It is just tradition. But you can take very good care of your wife. You can wash, cook... What do you need your mother for? She already brought you up. You as a father should play your role in the family. (Urban father, FGD)

#### Increasing tensions

In the survey the mothers were asked questions about their relationship with their mother-in-law and their partner. The mothers-in-law were given significantly lower scores than the partners on all questions (p < 0.001) (Table 3). The mothers ranked their mothers-in-law particularly low on the questions regarding trust and power. There were no significant differences between the responses from urban and rural mothers with respect to these questions.

**Table 3:** Comparison of mean scores in relationship questions regarding mother-in-law and partner

Empirical subgroups and individual questions	Mean score mother-in-law (95%CI)	Mean score partner (95%CI)
**'Trust'**		
**I share all the information I receive at the antenatal clinic with my partner/mother-in-law**	3.10 (2.68-3.51)	8.88 (8.62-9.15)
**I would share my secrets with my partner/mother-in-law**	2.68 (2.33-3.03)	7.59 (7.28-7.89)
**'Power'**		
**I need to do what my partner/mother-in-law wants me to do**	2.06 (1.73-2.39)	5.57 (5.23-5.91)
**I can only feed our infant in a way my partner/mother-in-law approves of**	1.34 (1.07-1.62)	3.19 (2.83-3.55)
**'Support'**		
**If I were sick and confined to bed my partner/mother-in-law would look after me**	6.40 (6.03-6.77)	9.10 (8.87-9.32)
**If my mother-in-law/partner treated me badly, I could trust my partner/mother-in-law to support me**	5.10 (4.73-5.47)	7.58 (7.30-7.87)

Paired t-test: all < 0.001.

All groups of informants in the qualitative interviews were familiar with the tensions and potential disagreements between daughter- and mother-in-law, but in the in-depth interviews, the majority of the daughters- and mothers-in-law stated that they had a good relationship.

She is fine; she is just like my own mother. (Urban mother, in-depth interview)

I regard her as my own daughter, since whatever bad things I say about her would reflect badly on my son too. (Urban mother-in-law, FGD)

However, during the FGDs several daughters- and mothers-in-law mirrored the well-known tensions between them. The obligations to pay respect and to obey the advice and demands of the senior woman came out strongly.

You need to follow her [the mother-in-law's] rules, because if you disagree there will be no love. (Rural mother, FGD)

Several mothers-in-law expressed common frustrations regarding the relationship between a mother-and a daughter-in-law:

There are some daughter-in-laws who do not listen to what you are telling them, they want to wander about and when you ask her to cook or do her responsibilities she says "ah, I would have preferred a husband whose parents are already dead. I am married to my husband, not you." You as mother-in-law, try to tolerate this but in the end you get tired. You decide to leave and let her live alone with her husband. (Urban mother-in-law, FGD)

During the discussions it was evident that it was the male partner who would have the final say if he was involved in their disagreements.

My mother has no right to make decisions in my house, its mine and my wife's. (Rural father, FGD)

#### Expecting authority

Mothers-in-law, in their position as elderly women and grandmothers, generally saw themselves as responsible for family health. By adhering to their customary defined commitments, mothers-in-law would expect their daughters-in-law to disclose information from the clinic to them.

When your daughter-in-law comes back from the hospital she has to tell you first what she was told there, since here she does not have a mother, you are her mother. Your role now is to give her more advice. (Urban mother-in-law, FGD)

Mothers in the survey rarely reported the mother-in-law as being responsible for decisions concerning health issues in the family (Table 2). The minority who did were almost all living in a rural area. The majority of mothers and fathers in the qualitative interviews stated that decisions concerning health issues were made by the couple, together. They were generally averse to involving the mother-in-law and appeared sceptical of her advice.

Me and my husband make the decisions. Sometimes my mother-in-law can say something, but if it is not good we do not listen. (Urban mother, in-depth interview)

The mother-in-law's experiences concerning infant feeding were deemed outdated by many and the mothers and fathers would rather follow advice given at the clinic.

I would not trust my mother too much in matters of feeding. Matters of breastfeeding are under my wife's care and my own mother is not involved at all. (Urban father, FGD)

However, some mothers, particularly those living in a rural area, stated that the mother-in-law was an influential person with great power over her daughter-in-law.

She can ask you to do something against your will and you have to listen to her because she is who she is. (Rural mother, FGD)

### The role of the mother-in-law in PMTCT

#### Attitude to the PMTCT programme components

Although mothers-in-law rarely escorted their daughters-in-law to the antenatal clinic, the majority knew about the PMTCT programme.

In the PMTCT clinic they advise the mothers on the importance of testing, to use condoms and to advise the husband to go for testing as well. If they are HIV positive they will receive medication. (Rural mother-in-law, FGD)

HIV testing for pregnant women, medication for HIV-infected mothers and delivery at a hospital were among the mothers-in-law perceived as important and beneficial and the participation in these components of the PMTCT programme was clearly supported. However, as guardians of customary infant feeding and of the survival and welfare of the infant, new infant feeding practises were met with greater resistance. The two infant feeding options for an HIV-infected mother, exclusive breastfeeding and exclusive replacement feeding, were both incongruent with the customary infant feeding practises in the Kilimanjaro region. Mothers-in-law encouraged and expected prolonged breastfeeding into the second year of life with early introduction of water and other nutrients.

#### Expectations to breastfeed

Breastfeeding was closely connected to the survival of the infant and was a very strong norm. If the daughter-in-law did not breastfeed, and in particular if she could not give a satisfactory explanation as to why she did not breastfeed, it would appear as incomprehensible and unacceptable to the mother-in-law who would commonly intervene.

I will not agree that my grandchild is denied breastfeeding without a good reason. (Urban mother-in-law, FGD)

If the daughter-in-law refused to give her an explanation she risked sanctions including being forced to breastfeed, not receiving postnatal care, being gossiped about or sent back to her husband in the middle of the confinement period:.

I will send her away to live with her husband since I can not live with a person who refuses to breastfeed her own child. (Rural mother-in-law, FGD)

However, a mother-in-law's anger was normally based on a concern for the health of her grandchild. Some would accept uncustomary feeding methods if they understood the rationale behind not breastfeeding.

I would probe until I know why she does not breastfeed, and if there is a reasonable reason then we will use alternative feeding methods. (Rural mother-in-law, in-depth interview)

Furthermore, mothers-in-law expressed great faith in health personnel. If they knew that clinical advice was behind not breastfeeding, they were unlikely to disagree.

I would completely disagree with her not breastfeeding unless she was given instructions by doctors. (Urban mother-in-law, FGD)

The mothers in the qualitative interviews would advice HIV-infected women who opted for exclusive replacement feeding, to inform their mother-in-law about their HIV status. The importance of communication with the mother-in-law was illustrated by the quote below:

If I had not shared with my in-laws that I am infected, I am sure that my mother-in-law would not want me to give formula. (Urban HIV-infected mother, in-depth interview)

#### Scepticism about exclusive breastfeeding

The concept and rationale behind exclusive breastfeeding was difficult for mothers-in-law to understand and accept, as it was incongruent with the information they had received when they themselves gave birth and breastfed:

Not giving water is very difficult. At the clinic they said that the baby should not be given water or milk other than the mother's. I was shocked! When we gave birth, the nurses used to encourage us to give baby water, saying that even the mother's milk causes thirst. And when we give water the baby drinks much. So, why should we not give them water? (Urban mother-in-law, FGD)

Some mothers-in-law stated that they would not have been able to remain passive if their grandchild was not given water. They would have been worried for the infant's health and would have given it water in the mother's absence.

You might make decisions if you see the baby is "drying up": I must protect my grandchild! If the mother is not there, I will give him water. (Urban mother-in-law, FGD)

Mothers and fathers were aware of the mothers-in-laws' scepticism about exclusive breastfeeding. A few rural mothers expressed a very submissive attitude towards their mother-in-law and would rather have given the infant water than opposing her. However, most of the mothers said that if they told their mother-in-law that it was clinical advice to breastfeed exclusively, she would accept it owing to her strong faith in health personnel. This was confirmed by some mothers-in-law.

I would not give water to him in his mother's absence, since his mother was told by a doctor not to give him water for the first six months. I am sure the doctors know better about these things. (Urban mother-in-law, FGD)

Mothers and fathers stated that if the partner was involved and accepted that his wife practised exclusive breastfeeding, he was likely to support her against his mother and have the final decision.

I would forbid my mother to give water because my wife should follow the doctor's advice. (Urban father, in-depth interview)

#### Disclosure and support

When asking mothers a hypothetical question concerning who they would have as their primary confidant if they were infected with HIV only 1.3% of the mothers, all residing in a rural area, would have chosen their mother-in-law (Table 2). The majority (60.4%) would have chosen their partner. When asking mothers the same hypothetical question in the qualitative interviews, they stated their husband as their first choice and secondly their own mother, sister or a close aunt. None of them mentioned their mother-in-law. She was often mentioned as the last person they would have told about a HIV positive test result. The mothers feared her reactions and did not trust her to be supportive.

I would not tell her because she would not understand me. She would think I brought it [HIV-infection] to her son and would send me away. (Urban mother, FGD)

Among the mothers-in-law, there were various views of how they would have reacted if their daughters-in-law had hypothetically been infected with HIV. Some said that they would have been obliged to support her.

I would support her and let her live with me. Where could I send her to, since she is my responsibility? (Urban mother-in-law, in-depth interview)

However, a minority admitted that they would have treated their daughters-in-law badly, and held her responsible for bringing HIV into the family. Two of the HIV-infected mothers, both of whom lived with their parents-in-law, reported to have experienced mistreatment by their mother-in-law. One of them was thrown out of the home after her husband died.

My in-laws chased me away, accusing me of infecting their son. (Rural HIV-infected mother, in-depth interview)

The following quote of the other HIV-infected mother illustrates the importance of a supportive partner:

My husband did not like the way his mother was treating me. So we decided to look for our own place and leave them alone. (Rural HIV-infected mother, in-depth interview)

## Discussion

This mixed method study about the role of the mothers-in-law in PMTCT service utilization and adherence to infant feeding guidelines in the Kilimanjaro region of Tanzania revealed that the mother-in-law perceived her role in confinement and child care as important. However, decisions concerning family health issues were usually made by the couple without involving the mother-in-law. The mother-in-law was generally supportive of the PMTCT services provided, but due to lack of knowledge about safe infant feeding and limited communication with the daughter-in-law, she could potentially be a barrier to safe infant feeding.

### Decreasing influence of mother-in-law

The mother-in-law saw herself as responsible for taking care of her daughter-in-law and paternal grandchildren. However, there appeared to be a discrepancy between the authority the mother-in-law expected and the authority that she received. In agreement with the findings from the Kilimanjaro region of the Pare population of Hollos *et al*, this study indicated a stronger conjugal union and a changed residence pattern in the wake of urbanisation [21]. Urban couples often lived by themselves and the physical distance from the mother-in-law seemed to decreased her influence [28]. In rural areas, where couples often lived nearer to the mother-in-law, she was perceived as more influential. Several of the rural mothers were already living together with their mother-in-law, which may explain why the custom of moving to live with their mother-in-law was not reported to be more common among the rural population as compared to the urban population. Overall, the influence of the mother-in-law seemed to be weaker in the current study than what has been reported elsewhere [3,8].

### Increasing strength of the marital union

Both the quantitative and qualitative data illustrated that mothers had much closer relationships with their partners than their mothers-in-law. Even if advice and support from the mother-in-law could be appreciated, couples usually stated that decisions concerning reproductive health issues were made among themselves without involvement of the mother-in-law. Therefore, the locus of decision making seems to have shifted towards the individual nuclear family [21]. According to Hollos *et al*, this development came in the wake of increased population density and the fragmentation of land holdings which precipitated a pattern of migration and a growing involvement in the cash economy by the majority of the present generation of younger males. Further, bride wealth is rarely paid and the choice of marital partners is increasingly in the hand of the young couple [21]. A large part of the women in this study were married. This might have increased the support received from their partners compared to a population where marriage is less common. However, these findings do not necessarily imply that women have gained a stronger position within their marriage or in relation to their mother-in-law. The findings could, however, suggest that the partner has gained a position closer to the one previously assigned to the mother-in-law. If the partner disagreed with his mother, and especially in cases where he did not depend on his parents for livelihood, he was able to disregard her opinion [29]. This coincides with the structural changes and facilitates a closer conjugal union. If these findings implies that the work directed at including the male partners in PMTCT has started to pay off is difficult to tell. In one of the other sub-studies of this work, exploring male involvement, we found that male partners were supportive of their wife attending the PMTCT programme, but there was substantial lack of active male partner involvement [17]. However, the prominent position of the male partner found in this study underscores the importance of continued efforts to include male partners in PMTCT [2,30].

### Lack of trust in mother-in-law

Mothers generally expressed a lack of trust in the knowledge and confidentiality of the mother-in-law, which seemed to be a barrier preventing her involvement in family health issues. Furthermore, mothers commonly feared the mother-in-law's reactions and did not trust her to be supportive if they had been HIV-infected. These fears could be justified as some of the mothers-in-law admitted that they would have treated their daughter-in-law badly if she had been HIV-infected. Nevertheless, the majority of mothers-in-law said that they would have supported an HIV-infected daughter-in-law had they been given the chance. These latter views expressed by the mothers-in-law could however reflect a "socially desirable" response to a hypothetical question about an HIV-infected daughter-in-law.

Mothers tended to describe a closer relationship to their mother-in-law in the in-depth interviews than in the FGDs. This could be due to lack of openness about personal negative experiences in the in-depth interviews or because the responses in the FGDs reflected collective attitudes to mothers-in-law rather than the actual experiences of the mothers.

### Infant feeding and the involvement of the mother-in-law

With regard to the PMTCT programme, the mother-in-law did not emerge as an obstacle to testing for HIV, to medication for mothers or children or to a safe delivery. However, if the daughter-in-law was HIV-infected and lived close to her mother-in-law, it could be difficult for her to follow the infant feeding advice without disclosing her HIV status, as reported in previous studies from the Kilimanjaro region [9,11]. Mothers-in-law would not accept an uncustomary infant feeding method for which they had not been given any rationale. Therefore, the lack of trust and open communication between daughter- and mother-in-law could become a barrier to safe infant feeding.

Ignoring the mother-in-law as an important partner in the health education given at clinics has undermined her potential powerful position in infant feeding. However, her support for breastfeeding and her concern for the health of the newborn could be utilized positively [3]. Taking into account their seemingly great confidence in the knowledge of health personnel, strategies to include the mother-in-law in the health education should be further explored. If the mothers-in-law were given the rationale behind the feeding method from health personnel, they would probably be more likely to accept it and not interfere with water and the like. Active involvement of grandmothers in nutrition education has been recommended previously [8], and in a study carried out in Senegal where grandmothers were included exclusive breastfeeding rates increased to above 90% [3]. This underscores the importance of building of good relationships between clinic staff and the community in general, and grandmothers influence in child rearing in particular.

### Methodological limitations

The concurrent mixed method design did not allow for information gained by one method to influence the next method as would had been the case if a sequential design had been used.

#### Truth value

The principal investigator was not conversant in the local languages. An interpreter was used when the in-depth interviews were conducted by the principal investigator. This might have created a distance between the interviewer and the interviewee. The FGDs were conducted by an experienced moderator trained in discussing sensitive topics. The principal investigator had a continuous discussion regarding responses and decided on the need for additional and supplemental information. Each FGD was transcribed and translated before the next FGD was performed so that these adjustments could be made if necessary. The principal investigator analysed qualitative data from transcripts that had been translated to English. Translation is always associated with various aspects of loss or modification of meaning; therefore, some of the original meanings might be lost. Despite this limitation, it was considered important that the principal investigator was able to take the lead since she knew her research objectives best.

Some of the questions asked during the qualitative interviews were hypothetical including how a mother-in-law would react to an HIV diagnosis in a daughter-in-law. We acknowledge that the answers given may have differed if actual experiences had been discussed.

Convenience sampling, selecting those most readily available, has the lowest credibility of the different qualitative sampling strategies [31]. In addition, familiarity with the main research assistant or the village leader may have affected the informants' willingness to participate. In some of the focus groups, several of the participants were familiar with each other, which may have inhibited openness when discussing sensitive issues.

A limitation of the quantitative study was the facility-based design which could have made it difficult for the mother to decline participation in the study and could have introduced a social desirability bias.

The use of the VAS scale could introduce bias if the mothers did not understand the scale, although all participants were given test questions in advance to prevent misunderstandings. Furthermore, a 0-10 scale could introduce bias by preferences for the numbers at the very end of the scale, 0 and 10, or in the middle of the scale, 5 [32]. However, this did not appear to be the case. The assessments are highly subjective, and it has been stated that the scale is most useful when looking at change within one individual and of less value in comparing across a group of individuals at one point of time [25]. Therefore, caution is required in the interpretation of the results.

#### Applicability

In our study 50% of the study population lived in rural areas, while in the Kilimanjaro region, about 80% of the population lives in rural areas, thus our selection of participants are not representative for the Kilimanjaro region.

Subsequent sampling of the mothers in the selected clinics may inhibit generalisation of the quantitative findings, as opposed to a randomised study. However, Moshi, in the Kilimanjaro region, is known for its high clinical attendance so it is likely that the sample is representative of Moshi pregnant women. But the area is known for a high educational level and high utilization of health services [10,20,33], so it might not be representative for the country as a whole.

## Conclusions

Decreasing influence of the mother-in-law and increased involvement of the male partner re-emphasised the importance of continued efforts to include the male partners in the PMTCT programme. Mothers were reluctant to involve the mother-in-law in infant feeding or to disclose their HIV status to her. The limited communication could be a barrier to healthy infant feeding practices, particularly for HIV-infected mothers. However, the mother-in-laws valued and respected health care workers. Thus, if they were given the rationale behind a feeding method they would be likely to accept it. Further research on the feasibility of including mothers- in-law in breastfeeding promotion programmes, so that they could potential become valuable supporters to safe infant feeding for HIV-infected and non-infected mothers alike, are recommended.

## Competing interests

The authors declare that they have no competing interests.

## Authors' contributions

All authors participated in the design and planning of the study: the field work was mainly conducted by EFF; the analysis and write-up was carried out mainly by EFF, KMM, TT, MMdP, SCL and IMSE. All of the authors read and approved the final manuscript.

## Authors' information

EFF is a medical doctor and PhD candidate. She has research experience from a qualitative infant feeding study in Zambia. KMM is associated professor at the Department of Nursing at Bergen University College. She is a nurse and a social scientist with a PhD in maternal-and reproductive health from the Kilimanjaro region. She has extensive experience from qualitative methods from research in Tanzania and Ethiopia. TT has a Master in African Linguistics and is a paediatrician and professor at the Centre for International Health at University of Bergen, with extensive experience from health related research in sub-Sahara Africa. MMdP is a nutritionist with a PhD in public health nutrition. She has extensive experience from mixed methods research from Tanzania, South-Africa and India. SCL is a nurse at School of Nursing at Muhimbili University College of Health Sciences in Dar es Salaam, Tanzania. She has a PhD in infant feeding and HIV from the Kilimanjaro region. IMSE is a medical doctor with a PhD in child health and nutrition and has experience from mixed methods research in Uganda.

## Pre-publication history

The pre-publication history for this paper can be accessed here:

http://www.biomedcentral.com/1471-2458/11/551/prepub
